# Fever—Its Pathology and Treatment by Antipyretics

**Published:** 1891-08

**Authors:** 


FEVER—ITS PATHOLOGY AND TREATMENT BY ANTIPYRETICS. Being an essay which was awarded the Royston Prize of Howard University, July, 1890: By Hobart Amory Hare, M. D. Sc., Philadelphia and London, F. A. Davis, Publisher.

I have read this little book with a great deal of interest. Hitherto the prescribing and administration of coal tar products as antipyretics have been done upon the "ipse dixit" of Knorr, the patentee, and his German sponsors, and the profession have gradually adopted them with even more eagerness than they have Koch's Remedy for Consumption. I have been amazed at the facility, and I might say the docility, with which the profession now-a-days welcome "New Remedies" with a patent stamped upon them.

Many physicians, however, have not permitted themselves to be led thus wise for the following reasons:

First. They look upon coal tar products as unstable, easily changed and resolved into poisons. Especially such is the case with antifebrine.

Second. "In the language of one of our most honored colleagues they consider it the duty of the profession to serve the art and not the trade of medicine, believing that honorable, legitimate medicine has no secrets to conceal and holds no remedy which is not the common heritage of the glorious brotherhood of the noble republic of science."

A careful perusal of this book will remove, in a great measure, the clouds that have shadowed this subject, yet the fact remains indisputable that who ever prescribes antipyrine, antifebrine, phenacetine et id omne genus, endorses a patent medicine. E. VAN G.

